# Editorial: Environmental risk factors in autism spectrum disorder

**DOI:** 10.3389/fpsyt.2022.978489

**Published:** 2022-08-23

**Authors:** Hideo Matsuzaki, Kohji Fukunaga

**Affiliations:** ^1^Research Center for Child Mental Development, University of Fukui, Fukui, Japan; ^2^Life Science Innovation Center, University of Fukui, Fukui, Japan; ^3^Department of CNS Drug Innovation, Graduate School of Pharmaceutical Sciences, Tohoku University, Sendai, Japan

**Keywords:** autism spectrum disorder, prenatal environment, inflammation, maternal immune activation (MIA), polygenic risk score

Autism spectrum disorder (ASD) is a neurodevelopmental disease defined by social impairments and repetitive behaviors. While ASD are highly heritable, several environmental risk factors (e.g., maternal infection exposure to drugs or toxicants, and brain inflammation) have also been suggested, although the underlying causes remain controversial. To shed more light on this, our Research Topic presents a broad range of insights to define the causal environmental factors in animal models and patients, and to answer the questions of how and what neuronal circuits are involved in each symptom including social impairments and repetitive behaviors. Specifically, this Research Topic included functional assessments how environmental factors correlate to ASD risk by using clinical study and animal models. This Editorial introduce following articles in the special issue on the environmental risk factors in ASD.

Firstly, three clinical study reports discussing some environment factors associated with ASD and their psychometric properties were introduced. Abelson et al. conducted a nested matched case-control study of children with/without ASD, and compared the use of antimicrobial therapy during the 3 months before conception or during pregnancy between mothers of cases and controls and used multivariate conditional logistic regression models to assess the independent association between maternal use of antimicrobials during pregnancy and the risk of ASD in their offspring. They concluded that the reduced risk of ASD associated with prenatal antimicrobials use only in the Jewish population suggest the involvement of other ethnic differences in healthcare services utilization in this association. Iwabuchi et al. investigated the associations between mothers' metabolic conditions, leptin concentrations in umbilical cord serum, and autistic symptoms among 762 children from an ongoing cohort study, and identified the umbilical cord leptin levels were associated with pre-pregnancy overweight, diabetes mellitus and SRS-2 scores in children. Although associations between maternal metabolic factors and autistic symptoms were not significant, these results imply that prenatal pro-inflammatory environments affected by maternal metabolic conditions may contribute to the development of autistic symptoms in children. Yoshikawa et al. were focusing on adverse childhood experiences (ACEs). In general, individuals with ASD have an increased risk of ACEs than typically developed (TD) children. They investigated the relationship between ACEs and microstructural integrity on frontal lobe-related white matter tracts using diffusion tensor imaging in 63 individuals with ASD and 38 TD participants, and suggested that an exposure to ACEs is associated with abnormality in the frontal lobe-related white matter in ASD.

Secondly, two animal model reports discussing some environment factors associated with ASD-like behaviors and brain phenotype were introduced. Kotajima-Murakami et al. examined whether exposure to the GABAA receptor antagonist picrotoxin causes ASD-like pathophysiology in offspring by conducting behavioral tests from the juvenile period to adulthood and performing gene expression analyses in mature mouse brains. They found that male mice exposed prenatally to picrotoxin exhibited a reduction of the social interaction in both adolescence and adulthood and showed a strong correlation between social interaction and enrichment of the “odorant binding” pathway gene module by using weighted gene co-expression network analysis (Kotajima-Murakami et al.). Their findings suggest that exposure to a GABAA receptor inhibitor during the embryonic period induces ASD-like behavior, and impairments in odorant function may contribute to social deficits in offspring. In other hands, it has been documented that the neuropeptide oxytocin (OXT) ameliorates core symptoms in patients with ASD. Matsuo et al. have recently reported that chronic administration of intranasal OXT reversed social and learning impairments in prenatally valproic acid (VPA)-exposed rats (1). They explored here molecular alterations in the hippocampus of rats and the effects of chronic administration of intranasal OXT, and clarified molecular profiling in the hippocampus related to ASD and improvement by chronic treatment with OXT (Matsuo et al.).

Finally, four review articles provide an interesting perspective focusing on maternal immune activation (MIA) resulting from bacterial or viral infection during pregnancy. Two of four are also focusing on role of interleukin-17A (IL-17A) on ASD etiology. Fujitani et al. examined the signaling pathways in both immunological and neurological contexts that may contribute to the improvement of autism spectrum disorder symptoms associated with maternal blocking of IL-17A and adult exposure to IL-17A, and suggested IL-17A antibodies may have ability to prevent ASD. Carter et al. focused on maternal immune activation (MIA) rodent models of ASD and reviewed the animal and human-based evidence indicating that IL-17A may mediate the observed effects of MIA on neurodevelopmental outcomes in the offspring. As severe acute respiratory syndrome coronavirus 2 (SARS-CoV-2) infection during pregnancy is a potent stimulator of the maternal immune response, authors state that this underscores the importance of monitoring neurodevelopmental outcomes in children exposed to SARS-CoV-2-induced MIA during gestation. Sato et al. reviewed evidence of the mechanisms by which environmental factors are related to ASD from three factor including prenatal drug exposure, parental aging, and MIA. Eve et al. reviewed articles to address the unexplored role that neuronal cell adhesion molecules may play in mediating inflammatory cascades that underpin neuroinflammation in ASD, primarily focusing on the Notch, nuclear factor-κB (NF-κB), and mitogen-activated protein kinase (MAPK) cascades.

To address the therapeutic approaches in ASD, we aim to define the causal environmental factors in animal models and patients. The goal of this Research Topic is to discover the novel therapeutic targets by definition of the common pathways and common environmental risks in human and animal models. As combination of environmental and genetic factors are believed to contribute to ASD pathogenesis, the perspective arisen from the articles may contribute to the development of preventive and therapeutic interventions for ASD. However, further research is needed to determine whether these risk factors are specific to ASD. Since the readers are now interesting in the polygenic risk score related to ASD, we expected submission focused on the connection between the polygenetic risk score and environmental risk. However, there was no such paper this time unfortunately. Further technological developments will deepen our understanding of the role of environmental factors in ASD etiology.

## Author contributions

HM prepared the manuscript. All authors contributed to the article and approved the submitted version.

## Conflict of interest

The authors declare that the research was conducted in the absence of any commercial or financial relationships that could be construed as a potential conflict of interest.

## Publisher's note

All claims expressed in this article are solely those of the authors and do not necessarily represent those of their affiliated organizations, or those of the publisher, the editors and the reviewers. Any product that may be evaluated in this article, or claim that may be made by its manufacturer, is not guaranteed or endorsed by the publisher.
